# Prevalence of coronaviruses in European bison (*Bison bonasus*) in Poland

**DOI:** 10.1038/s41598-024-63717-1

**Published:** 2024-06-05

**Authors:** Magdalena Larska, Jarosław Tomana, Michał K. Krzysiak, Małgorzata Pomorska-Mól, Wojciech Socha

**Affiliations:** 1 Department of Virology, National Veterinary Research Institute, Puławy, Poland; 2 Veterinary Clinic, Pszczyna, Poland; 3 Sub-Department of Parasitology and Invasive Diseases, Veterinary Faculty, University of Life Sciences, Lublin, Poland; 4 Department of Preclinical Sciences and Infectious Diseases, Faculty of Veterinary Medicine and Animal Science, University of Life Sciences, Poznan, Poland

**Keywords:** Coronaviruses, BCoV, European bison, Seroprevalence, PCR, Sanger sequencing, SARS-CoV-2, Ecological epidemiology

## Abstract

Coronaviruses have been confirmed to infect a variety of species, but only one case of associated winter dysentery of European bison has been described. The study aimed to analyze the prevalence, and define the impact on the species conservation, the source of coronavirus infection, and the role of the European bison in the transmission of the pathogen in Poland. Molecular and serological screening was performed on 409 European bison from 6 free-ranging and 14 captive herds over the period of 6 years (2017–2023). Presence of coronavirus was confirmed in one nasal swab by pancoronavirus RT-PCR and in 3 nasal swab samples by bovine coronavirus (BCoV) specific real time RT-PCR. The detected virus showed high (> 98%) homology in both RdRp and Spike genes to BCoV strains characterised recently in Polish cattle and strains isolated from wild cervids in Italy. Antibodies specific to BCoV were found in 6.4% of tested samples, all originating from free-ranging animals. Seroprevalence was higher in adult animals over 5 years of age (*p* = 0.0015) and in females (*p* = 0.09). Our results suggest that European bison play only a limited role as reservoirs of bovine-like coronaviruses. Although the most probable source of infections in the European bison population in Poland is cattle, other wild ruminants could also be involved. In addition, the zoonotic potential of bovine coronaviruses is quite low.

## Introduction

The European bison (*Bison bonasus*) is the largest extant mammal is Europe with bulls reaching almost 2 and 3 m of height and length, respectively, and 900 kg in body mass. The European bison became the subject of the prehistorical paintings and sculptures found in caves in Spain (Altamira) and France (Tuc d’Audoubert). The original range of *Bison bonasus* covered most of the continent. As the second millennium got underway, European bison were still present in the extensive forests covering Europe. However, as the woodland was replaced by field cultivation, the habitat began to shrink. The process of gradual decline towards extinction had begun as early as in the eleventh century, and was maintained without reverse up to the nineteenth century in the last refuge for the species—Białowieża Primeval Forest. The survival of the European bison in this one place was determined not only by the specific characteristics of the untouched for centuries natural forest, but also by the care and protection efforts of the kings of Poland and Russian tsars. After World War I, European bison became extinct in the wild, when the last animal was killed in the Białowieża Forest in 1919. They survived in a few zoos and enclosures in Europe, but thanks to an international breeding program in the 1920s and 1930s, the population began to increase, firstly, as a herd established back in Białowieża, which became a cradle for the present European bison population reaching more than 10,000 individuals across Europe^1^ while, the animals are being reintroduced in the distant areas such as Spain, Portugal and Romania, knowledge of health threats to the species needs to be continuously researched and updated.

**Figure 1:**
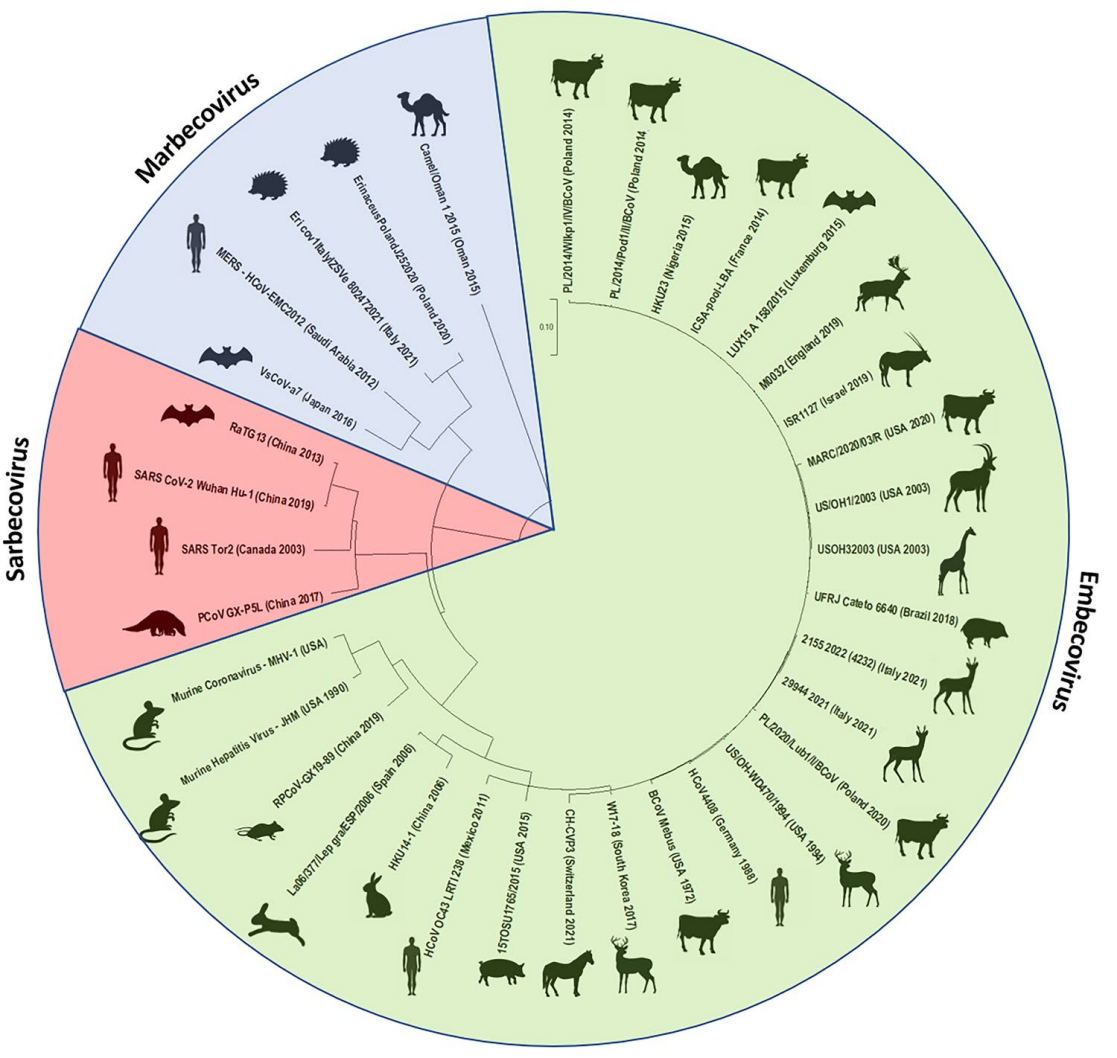
Phylogenetic relationship of selected members of Betacoronavirus genus infecting wild,related domestic mammals, and human. Country of origin and year of detection of selected strains are included in the brackets. Three subgenus are distinguished: Embecovirus (green), Sarbecovirus (red) and Marbecovirus (blue). Phylogenetic tree was constructed by neighbour-joining method based on the RdRp gene sequences of coronaviruses available in GenBank, using MEGA11 software.

Coronaviruses (CoVs) are enveloped RNA viruses belonging to the Coronaviridae family, order Nidovirales. As recent years have shown, they have a huge epidemic potential, with high genetic variability, adaptative capacity, and potential for interspecies transmission. They are capable of infecting a wide variety of mammalian and avian species causing respiratory, enteric, hepatic, and in rare cases also neurological diseases. Coronaviruses’ high diversity is associated with low fidelity of viral RNA polymerase resulting in a high rate of mutations, large genome size, and high frequency of homologous recombination. As a result, CoVs could easily adapt to new hosts and ecological niches. The importance of this potential of CoVs has become evident in the last decades as the spread and adaptation of three coronaviruses of animal origin to humans led to outbreaks of severe acute respiratory syndrome coronavirus (SARS-CoV), Middle East respiratory syndrome coronavirus (MERS-CoV) and the pandemic of SARS-CoV-2^2,3^. Susceptibility of various wild and domestic animals to SARS-CoV-2 infections has been evaluated in several studies based on analysis of Angiotensin-converting enzyme 2 (ACE2) surface receptor of the host, study of virus prevalence in collected field samples and experimental infections^4,5^. The aforementioned ACE-2 receptor is responsible for coronavirus jumping between different hosts^4^, however little is known about interspecies transmission, except for the epidemic strains like SARS-CoV-2^6^. Spill-over events of SARS-CoV-2 from humans affected far more species than were identified at the start of the outbreak (pangolins and bats). The susceptibility to the virus was confirmed in over twenty animal species including ferrates, felines, and canids^7–10^. While the deer-to-deer and deer-to-human transmission of SARS-CoV2 was confirmed in white-tailed deer (WTD; *Odocoileus virginianus*) in North America, the involvement of European cervids in the pandemic has, however, been dismissed due to the lack of evidence of their exposure^11–18^. Exposure to Alpha, Delta, and Omicron variants was only shown in some individual fallow and red deer in the suburban areas of Madrid, Spain^19^, suggesting some possible spill-over events, however, rather accidental, not relevant for maintaining the virus in the environment. The ability of intraspecies transmission was also suggested based on the high homology of Bovine Coronavirus (BCoV) strains from cattle and BCoV-like *Embecoviruses* described in non-domesticated wild ruminants (Fig. 1)^3,20^, although such events have not been proven in field studies yet. Transmission between farmed and wild ruminants and vice versa may facilitate the persistence of this virus in nature, the recurrent emergence of epizootics, and its continuous evolution^20^.

The available data on coronavirus infections in wildlife in Poland is limited, as the only wild mammalian species in which the presence of CoVs was confirmed were hedgehogs and bats^21,22^. A small study on SARS-CoV-2 exposure in red deer in North-Eastern Poland did not confirm any possible involvement of this species in the transmission of the pandemic virus, how it was shown for farmed mink^23^, and incidental exposure of pets^14,24^. BCoV-like viruses were described in Sable antelope, giraffe, sambar deer, alpaca, waterbuck, elk, roe deer, and even WTD, described to be susceptible to SARS-CoV-2^3,15,20,25,26^ (Fig. 1).

The studies on European bison as a reservoir of pathogens threating domestic animals and humans suggest mostly that the wild species exposure is due to the spillover from the synanthropic counterparts, however, no direct evidence on intraspecies transmission exists^27^. Due to their size, European bison are an ideal food source first for blood-sucking insects and second for scavengers and top predators. European bison is also considered as umbrella-species contributing to the protection of flora and small fauna and enriching biodiversity. We have shown that they are an important reservoir or are responsible for the persistence of emerging and re-emerging arboviruses in the environment^28,29^, which similarly to coronavirus^30^ are considered climate-sensitive infections, meaning that they may be more seasonal, and frequency, rather than density-dependent. Previously, some herds of European bison in the Bieszczady mountains infected with *Mycobacterium caprae* may have contributed to the contamination of the environment and the exposure of other wild species including deer, wild boar and wolves^31^, while tuberculosis was successfully eradicated by selective culling in European bison in Bieszczady. The relevance of climatic parameters to the incidence of BCoV infection in cattle is reflected by the name of the disease it causes—winter dysentery (WD). The risk associated with coronavirus infections should also be considered important for the protection of European bison, as the species face the new challenges of the modern world related to environmental and anthropogenic changes. Poland is the homeland of the largest free-living population of European bison, whose number has increased significantly in recent years. The population size has exceeded the limits of the habitat capacity of large forest complexes such as the Białowieża Primeval Forest, resulting in the expansion of European bison into agricultural areas. In addition to the conflicts and losses associated with the damage caused by these large mammals, the possibility of direct (pastures) and indirect (manure fertilization) contact with livestock and humans, and thus the potential transmission of pathogens, is also increasing. Potential transmission of viruses between cattle and European bison is supported by results of recent studies on the seroprevalences of multiple viral pathogens known to infect cattle including bovine herpesvirus 1 (BoHV-1), bovine respiratory syncytial virus (BRSV), bovine adenovirus (BAdV), bovine viral diarrhea virus (BVDV) or bovine parainfluenza virus-3 (BPIV-3)^27,32^. The European bison protection strategy involves creating more, smaller populations by translocations into new areas and countries such as Spain, Romania, Netherlands, and Portugal, thus creating potential risk of pathogen transmission. Despite that the species’ status by International Union for Conservation of Nature (IUCN) Red List has been changed from vulnerable to near threatened, the species remains endangered, also due to the low genetic diversity of the entire global European bison population, which may lead to impaired resistance to health hazards and resilience to changing environment. It urges the necessity for surveillance and control strategies as a tool for the conservation and prevention of epidemiological risks.

The epidemiological status of European bison remains poorly recognized as a single case of winter dysentery associated with Bovine-like CoV was described in an individual European bison housed in a zoo in Korea^25^. Additionally, the susceptibility of European bison to SARS-CoV-2 remains unexplored as only one individual was included in a recent serosurvey of wild ruminant screening in Germany, which also proved to be a negative^18^. Therefore, the study aimed to evaluate the occurrence coronaviruses in the European bison as it can represent unrecognized health risks that could affect the conservation program.

## Results

### Coronavirus infections in European bison

**Figure 2:**
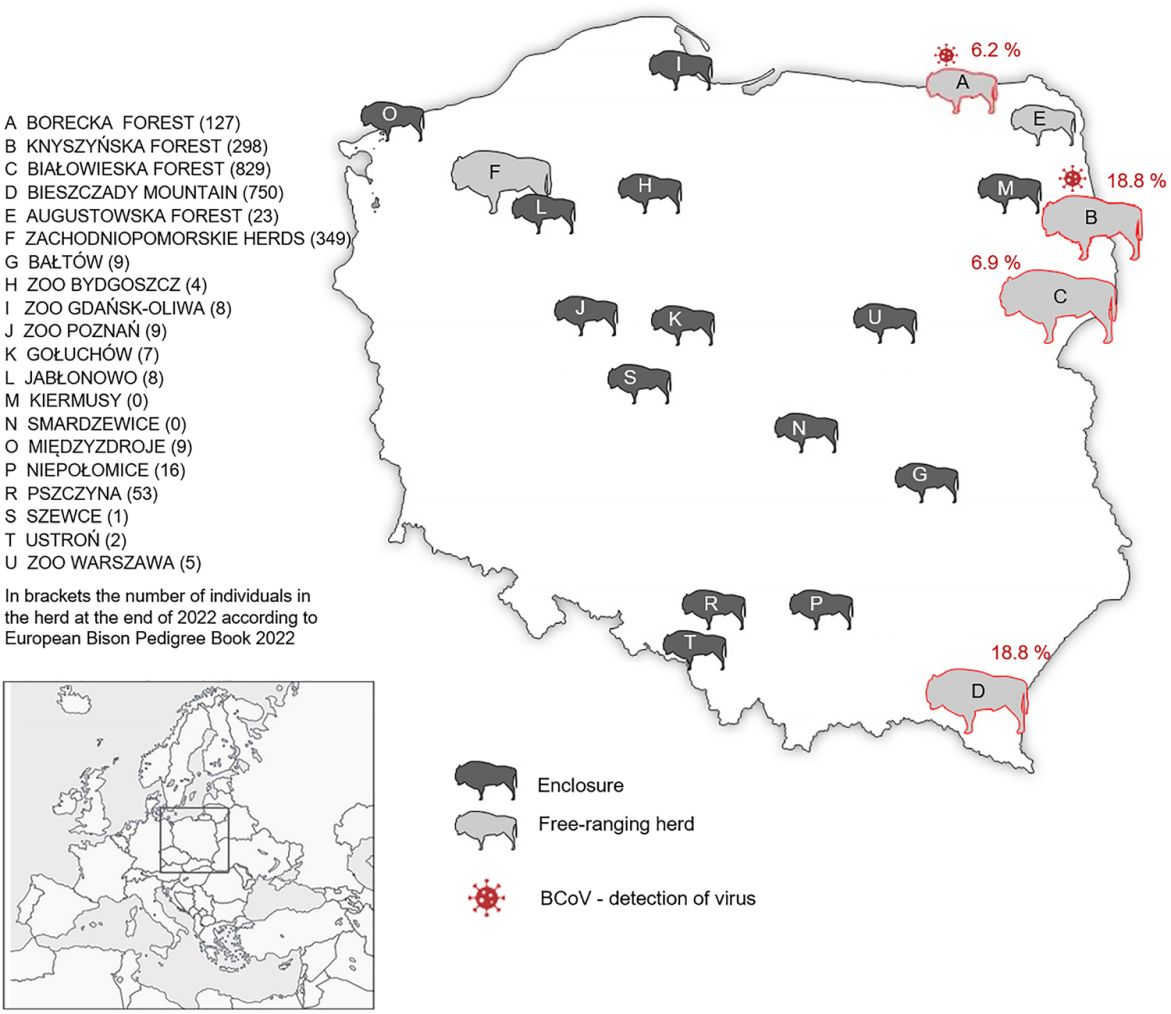
Map of the free-ranging populations (light grey silhouette) and enclosures (dark grey silhouette), where tested European bison originated from. Two non-existing in 2022 captive herds: Kiermusy (M) and Smardzewice (N) were sampled between 2018 and 2021 (M) and in 2018 (N), that is, until they were closed down. Seropositive herds were red framed, while red virus sign indicated locations where coronavirus infected individuals were detected. The size of the silhouette on the map corresponds approximately to the herd /population size.

The virus was detected in only one (0.3%) nasal swab sample collected from European bison originating from Borecka forest in 2019 in pancoronavirus RT-PCR. The result was confirmed by BCoV specific real-time RT-PCR, together with two other positive swab samples, collected from two individuals from the free-ranging Knyszyńska forest population in 2019 and 2020 (Fig. 2). Two of the animals the age of 7 and 13 years were healthy individuals chemically immobilized for other purposes. While one animal from Knyszyńska forest was 15-year-old bull sampled *post-mortem* after a traffic accident. The death of the above-mentioned animal occurred as a result of multi-organ damage resulting from severe trauma, no lesions indicative of a respiratory infection were found. All fecal samples were negative for the presence of coronavirus in both pancoronavirus RT-PCR and real-time BCoV tests.

**Figure 3:**
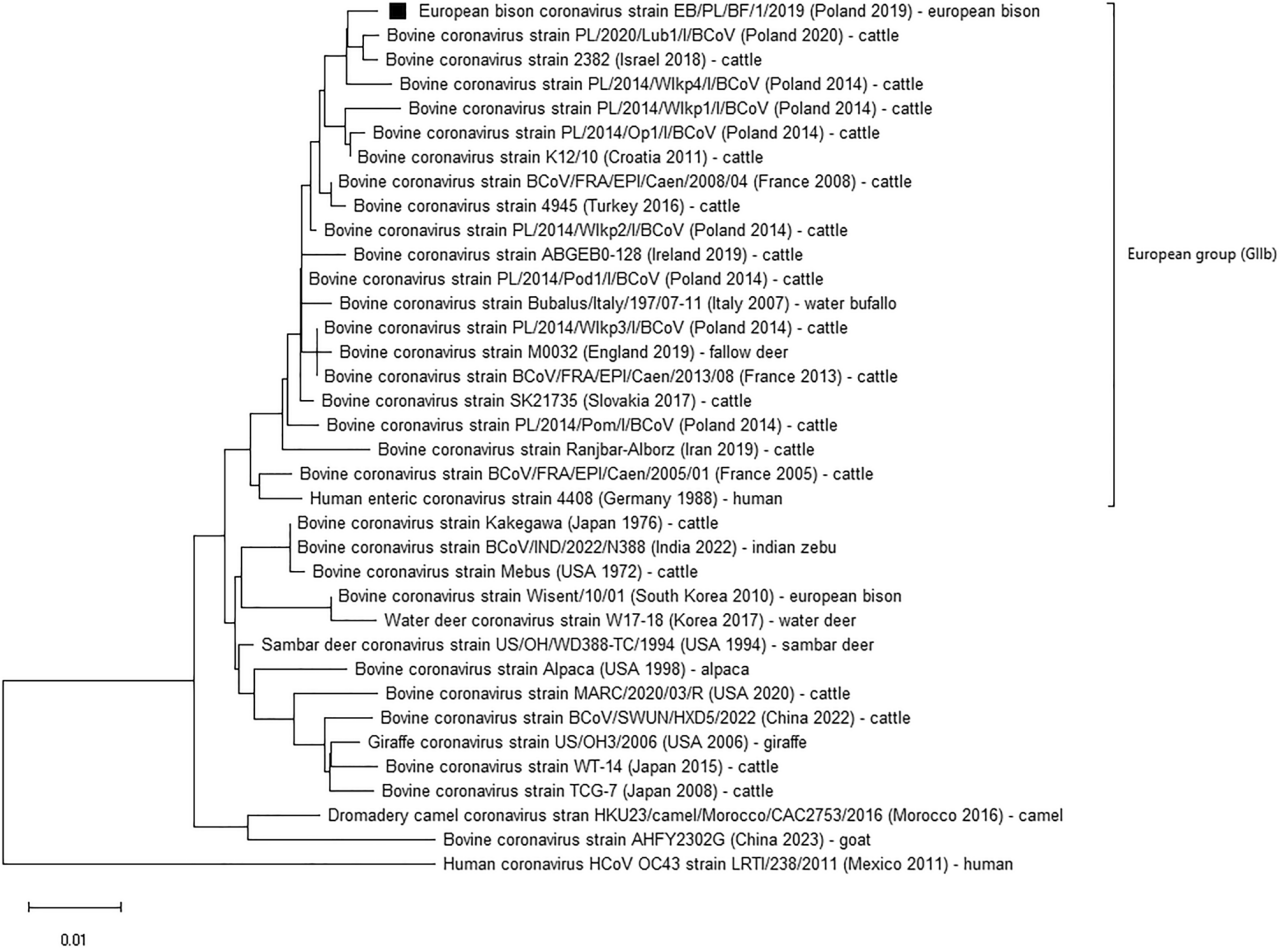
Neighbour-joining phylogenetic tree was constructed using a 601-nucleotide-long fragment of the gene encoding the spike protein of Betacoronaviruses with Human coronavirus HCoV OC43 used as an outgroup ^54^. Sequence acquired in this study is marked by black square. In brackets the European (GIIb) phylogenetic group as described by Shin et al. 2022 ^37^ was distinguished. Data about the country of origin, date of sample collection and hosts are included next to the name of each strain.

**Figure 4:**
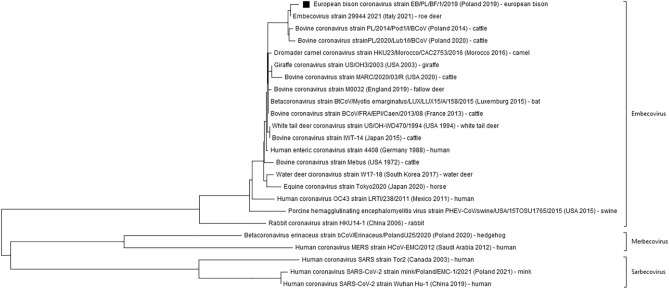
Neighbour-joining phylogenetic tree was constructed using a 432 nucleotide-long fragment of the gene encoding the RNA-dependent RNA polymerase (RdRp) of Betacoronaviruses with Human coronavirus SARS-CoV-2 used as an outgroup ^54^. Sequence acquired in this study is marked by a black square. In brackets 3 subgena of the Betacoronavirus genus were distinguished. Data about the country of origin, date of sample collection, and hosts are included next to the name of each strain.

Partial sequences of S and RdRp genes of one coronavirus strain isolated from European bison were aligned with sequences of previously identified coronaviruses isolated from cattle, human, and other species. Identified coronavirus strain named EB/PL/BF/1/2019 showed high homology of the nucleotide sequence in RdRp (98.6–99.5%) and Spike genes (98.3–99.3%) fragments to recently identified Polish strains of BCoV isolated from cattle^33^. High genetic homology in the RdRp gene was also found in coronavirus strains, isolated from roe deer in Italy in 2021 (99.7%) and in the Spike gene from strains isolated from cattle in Israel in 2018 (99.3%) and in Slovakia in 2017 (99.0%)^15,34,35^(Fig. 3, Fig. 4).

### Serological evidence of BCoV exposure in European bison

**Figure 5:**
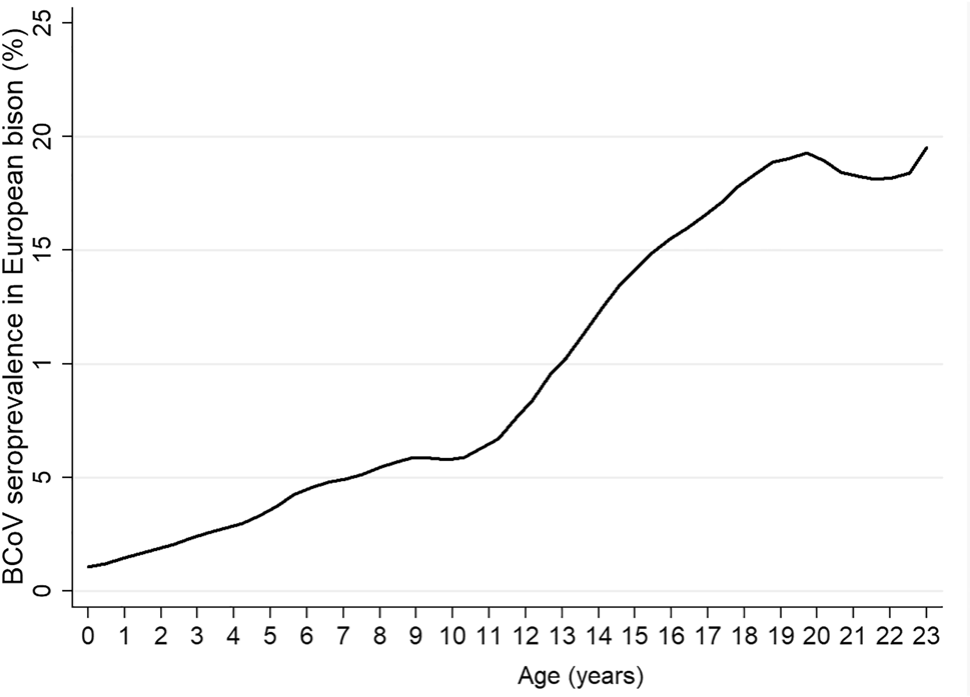
The local polynomial smoothed line represents BCoV seroprevalence by European bison age.

According to ELISA results, 26 out of 409 (6.8%) European bison had antibodies to BCoV. The antibody levels with regard to the semi-quantitative gradation of the test (+ to +  +  + +) were almost equally distributed among the positive serum. The highest seroprevalences were observed in the free-ranging populations, while none of the captive European bison had BCoV antibodies (Table 1). That could explain the higher seroprevalences in the selectively eliminated due to the poor health conditions and dead in accident animals, which mostly concern the free-ranging European bison. According to those results, the exposure to BCoV increased along with the age of the animals (Table 1, Fig. 5).

**Table 1:** Association between individual and herd-level parameters and Bovine coronavirus (BCoV) seroprevalence in European bison.

Variable	*n* positive/*N*	Seroprevalence (%)	95% CI^a^	χ^2^	*P*
Origin				32.2	0.04
Bałtów	0/6	0	0–46.0^c^		
Białowieska forest^b^	9/131	6.9	3.2–12.6		
Bieszczady mountains^b^	9/48	18.8	8.9–32.6		
Gołuchów	0/11	0	0–28.5^c^		
Jabłonowo	0/17	0	0–19.5^c^		
Kiermusy	0/7	0	0–40.9^c^		
Międzyzdroje	0/4	0	0–60.2^c^		
Niepołomice	0/13	0	0–24.7^c^		
Pszczyna	0/68	0	0–5.3^c^		
Augustowska forest	0/3	0	0–0-70.8^c^		
Borecka forest	2/32	6.2	0.8–20.8		
Knyszyńska forest	6/32	18.8	7.2–36.4		
Smardzewice	0/9	0	0–33.6^c^		
Szewce	0/1	0	0–97.5^c^		
Ustroń	0/3	0	0–70.8^c^		
Zachodniopomorskie herds	0/11	0	0–28.5^c^		
Zoo Bydgoszcz	0/1	0	0–97.5^c^		
Zoo Gdańsk	0/3	0	0–70.8^c^		
Zoo Poznań	0/6	0	0–45.9^c^		
Zoo Warszawa	0/3	0	0–70.8^c^		
Age group				10.6	0.005
Calves (≤ 1 year)	0/74	0	0–4.9^c^		
Youth (2–3 years)	2/74	2.7	0.3–9.4		
Adults (≥ 4 years)	23/242	9.5	6.1–13.9		
undefined	1/19	5.2	0.1–26.0		
Sex				2.8	0.09
Females	16/187	8.6	5.0–13.5		
Males	10/222	4.5	2.2–8.1		
Population type				23.6	< 0.001
Free-ranging	26/221	11.8	7.8–16.8		
Captive	0/188	0	0–1.9		
Sanitary status				11.4	0.01
Healthy (immobilized)	8/233	3.4	1.5–6.6		
Selectively eliminated	12/109	11.0	5.8–18.4		
Fallen	3/51	5.9	1.2–16.2		
Accident	3/16	18.8	4.0–45.6		

^a^binomial exact 95% confidence interval; ^b^included free-ranging and captive European bison; ^c^one-sided 97.5% confidence interval.

## Discussion

The current study is the first one to identify and characterise coronavirus circulating in European bison, indigenous to Europe free-roaming species of the *Bison* genus, Bovidae family. As expected, the virus detected belonged to a BCoV-like *Embecovirus* group of the *Betacoronavirus* genus. The low prevalence (1%) associated with viral shedding by nose only suggests that the strain circulating in European bison has a respiratory rather than enteric tropism^36^, however, CoV infections in the European bison population are incidental. Coronavirus-positive animals were detected by both pancoronaviral (1 sample) and BCoVs specific (3 samples) PCR reactions. While the number of positive results were different this should probably be associated with higher sensitivity of real-time PCR, as positive samples with high Ct values (> 35) in BCoV specific test were negative in pancoronavirus specific PCR. The fact that all positive samples were detected by BCoV-specific assays pointed on BCoV-related virus as a source of infection. This was further confirmed by sequence analysis of the one coronavirus positive sample isolated from European bison originating from the free population of Borecka forest. It showed 98.6 to 99.3% nucleotide sequence homology of the Spike protein encoding fragment and 98.6 to 99.7% in the RdRp gene to Polish strains of BCoV isolated from cattle^33^. Very high homology (over 99.5%) was also observed with RdRp sequences of 29944_2021 Embecovirus strain isolated in 2021 from Italian roe deer. All these strains could be assigned as related European lineage (or GIIb group as assigned by Shin et al. 2022^37^) of BCoV, composed of strains isolated from various ruminants such as cattle, fallow deer, roe deer, or buffalo, in the last two decades. These results are in line with previous studies on coronavirus infection in captive and non-captive wild ruminants such as sambar deer, white-tail deer, water deer, antelope, or giraffes. Betacoronaviruses isolated from those species shared a high degree of homology to local strains of Bovine coronavirus with no species-specific mutations observed^38,39^. A similar observation was made in Korea for the only coronavirus isolated till now from a European bison kept in a zoo^25^. Two possible explanations were proposed for the lack of species-specific CoVs among ruminants. First, that new host adaptation of CoVs might be defined by a combination of substitutions scattered throughout the whole genome that could not be easily distinguished based on phylogenetic analysis of partial genome sequence, and second—that no adaptation changes are needed for CoV interspecies transmission between ruminants^38^. The second explanation was additionally supported by a large-scale study on the evolution of the BCoV genome that showed only limited signs of host or tissue adaptation^40^. As the CoV isolate acquired in our study was equally similar to BCoV strains isolated from cattle and roe deer it is not possible to unequivocally establish the original source of infection. It is known that European bison and various species of wild cervids such as red deer are present in the same habitats in Poland^41^ and previous studies showed that deer species could be a source of CoV infection for other free-living ruminants (ex. sambar deer for waterbuck)^40^. The evidence that BCoV is not endemic in the European bison population and that the infection spilled out from other ruminants (cattle or cervids) is supported by the fact that we solely found infected individuals in free-ranging herds, not kept in enclosures. Further, it is more probable that the transmission originated from cattle, since our recent, yet unpublished study, showed that BCoV prevalence in Polish wild cervids is close to 0%. Whereas, our previous studies have confirmed that BCoV is highly prevalent in Polish unvaccinated cattle, with 72.6% antibody positives and 10.5% virus shedding by respiratory route individuals sampled between 2014 and 2015^33^. BCoV infection is more frequent in younger cattle and it is associated with bovine respiratory complex (BRD) in calves^33^. Coronavirus was detected in one-third of calves affected by BRD in the country, indicating its importance in the complex aetiology of the disease^42^. This contrasts with low (6.8%) BCoV seroprevalence among European bison and a strong correlation between age and the serological status with no seropositives among the youngest animals. Moreover, the lack of maternal antibodies to European bison calves below 6 months of age supports the rather incidental infection in the species unlike Polish cattle, which showed over 80% seroprevalence of this group of calves^33^. BCoV seroprevalence differed between herd sizes, with the lowest 50% infected in the smallest barns under 77 head which may represent those with which free-ranging European bison populations come into contact on pastures^33^. However, our results do not corroborate the observations of the results of a questionnaire among cattle breeders, which were asked to assess the quality of their cattle’s contact with European bison^43^. On the basis of their opinion the highest risk for virus transmission by direct or indirect contact or sharing pastures between European bison and cattle should be considered in in the area of Białowieża Forest, which was not supported by the relatively low BCoV seroprevalence among the free-ranging populations in our study^43^. However, the higher exposure of European bison may be explained by the higher density of cattle and their breeding in extensive conditions and more frequently grazed.It was supported by the number of seropositive (15 out of 26) and BCoV infected (2 out of 3) European bison, which originated from the Podlaskie region, a part of Poland with the highest cattle density per 100 ha of agricultural land (95.9 animals per 100 ha^44^). Moreover, one of the highest (over 80%) BCoV seroprevalences was previously reported in the cattle from this area^33^. The direction of transmission from cattle to European bison, has already been concluded for several endemic bovine pathogens^27,32^, which may suggest that this recently booming in numbers and expanding geographically population could threaten the very conservation of the species itself. European bison are often considered as an additional epidemiological risk to livestock^43^. Therefore, efforts to monitor the health threats should be undertaken, including at the destinations prior to European bison translocations. Although respiratory lesions are the most commonly observed in European bison^45^, there is no evidence that they may be related to coronavirus infection, as we only found BCoV shedding in individuals not displaying them. However, despite this, the highest seroprevalence was observed for European bison eliminated for health reasons and those that died in accidents, thus possibly co-infections with coronaviruses contribute to their overall poorer health condition^46^. Similarly, there is little evidence of clinical BCoV infections in other wild ruminant species^39,46,47^. Previously, it was shown that ruminant species, such as wild-tailed-deer could not only transmit SARS-CoV-2 but also be clinically affected by COVID-19^11,48,49^. In that case this was confirmation of previous theoretical studies, suggesting WTD vulnerability to SARS-CoV-2 basing on high degree of homology of ACE2 receptor, crucial for viral entry, with the human one^50^. While similar analysis of ACE2 receptor has not been done for European bison, it was previously shown that degree of this homology is lower in case of ACE2 receptor of closely related American bison^51^. Therefore, the lack of SARS-CoV-2 infections among European bisons tested in our study should not be surprising even though some close encounters with human might have occurred, especially in enclosures.

The free-ranging European bison population in Poland lives mainly in forested areas. The constant increase in the population of free-ranging European bison leads to an increase in animal density and migration of some individuals outside the previously occupied forest habitats and staying in agricultural areas (arable fields, meadows). Factors such as the increasing density of free-ranging European bison in a given area and the increase in the frequency of direct or indirect contact between them and farm or wild animals may pose a potential risk of an increase in the number of CoV infections in European bison. Currently, it is impossible to explain whether the spread of the CoV virus in European bison encounters obstacles to the adaptation of the virus to a new host or whether the spread is limited by too low frequency of contact between animals. In summary, to fully elucidate whether other wild ruminants or cattle play a more important role in the spread of CoVs among European bison, further serological and virological studies on populations of those animals in Poland are needed. Nevertheless, coronavirus infection in Polish European bison seems to be rare and probably has little effect on the health status of the population.

## Methods

### Samples

Samples were collected from 409 European bison (*Bison bonasus*) including healthy (immobilized for other purposes such as translocation or GPS collar placement); selectively eliminated due to poor health condition; fallen (found dead); and dead in an accident between 2017 and 2023. Serum samples were collected from each animal, along with either fecal samples, nasal swabs, or both. As a result, in total 284 nasal swabs, 354 fecal samples and 409 serum samples were included in the study. Samples originated from 221 free-ranging and 188 captive animals from 20 main populations including six free-ranging from all over the country. Among those tested 222 were males and 187 females. The age of the tested animals ranged from 1 day to 22 years.

### RNA extraction

Collected fecal samples were diluted in PBS to 10% solution and homogenized using Fastprep-24 5G™ (MP Biomedicals, Irvine CA, USA) homogenizer. Viral RNA was extracted from 140 µL of nasal swab and fecal homogenate samples using a QIAamp Viral RNA Mini kit (Qiagen, Hilden, Germany) according to the manufacturer’s guidelines. Ribonucleic acid was eluted in 50 µL of an elution buffer and stored at −70 °C.

### Pancoronavirus RT-PCR

Pancoronavirus RT-PCR was run using previously described primers specific to the RdRp gene^52^. The reaction was run in two steps. In first step, 25 µL reaction mix was used composed of 7 µL of water, 12.5 µL of 2 × reaction buffer, 1 µL of SuperScript III RT/Platinum Taq enzyme Mix (ThermoFisher Scientific, Carlsbad, USA), 1 µL of both Chu-RdRp-N1-F forward (10 µM) and Chu-RdRp-N1-R reverse (10 µM) primers, and 2.5 µL of RNA. In the second step 25 µL of reaction mix was used, comprising 17.5 µL of water, 2.5 µL of 10 × reaction buffer, 0.5 µL of Jumpstart AccuTaq™ LA DNA Polymerase (Sigma Aldrich, St Louis, USA), High fidelity Taq enzyme, 1 µL of both Chu-RdRp-N2-F forward (10 µM) and Chu-RdRp-N2-R reverse (10 µM) primers, 0.5 µL of dNTPs mix and 2 µL of DNA product from first reaction. Reaction conditions of first amplification included 30 min reverse transcription in 50 °C, 15 min incubation in 95 °C and 45 cycles of amplification, each consisting of 30 s denaturation in 95 °C, 30 s of annealing in 55 °C and 1 min elongation in 72 °C. The reaction was finished with 10 min incubation at 72 °C. For nested PCR, the reaction consisted of 10 min incubation at 95 °C, 40 cycles of amplification, with 30 s denaturation at 95 °C, 30 s of annealing at 48 °C and 45 s elongation at 72 °C and 4 min of final incubation in 72 °C. As a positive control, the BCoV S379 Riems strain was used. 5 μL of PCR product of the second reaction was used for visualization by electrophoresis on the 1.5% agarose gel. A sample was regarded as positive if a band of the expected 440 bp size was visible on the gel. Sequences of primers used for each step of the reaction are presented in Table 2.

**Table 2:** Primers and probes used for Coronaviridae, bovine coronavirus (BCoV) and mammal β Actin gene fragment as internal control amplification detection.

Target	Primer/probe	Sequence (5′–3′)	Amplicon size (bp)	Gene/protein	Reference
Pancoronavirus	Chu-RdRp-N1-F	GGKTGGGAYTAYCCKAARTG	602	RdRp	^52^
	Chu-RdRp-N1-R	TGYTGTSWRCARAAYTCRTG			
	Chu-RdRp-N2-F	GGTTGGGACTATCCTAAGTGTGA	440		
	Chu-RdRp-N2-R	CCATCATCAGATAGAATCATCAT			
BCoV	BCoV-F	CTGGAAGTTGGTGGAGTT	85	M/matrix	^53^
	BCoV-R	ATTATCGGCCTAACATACATC			
	BCoV-Pb	FAM-CCTTCATATCTATACACATCAAGTTGTT-BHQ1			
	Sp1	CTTATAAGTGCCCCCAAACTAAAT	622	S/spike	
	Sp2	CCTACTGTGAGATCACATGTTTG			
β Actin	ACT-1005-F	CAGCACAATGAAGATCAAGATCATC	130	bACT	^56^
	ACT-1135-R	CGGACTCATCGTACTCCTGCTT			
	ACT-1081-HEX	HEX-TCGCTGTCCACCTTCCAGCAGATGT- BHQ1			

*BCoV* bovine coronavirus, *FAM* fluorescein amidite, *ACT* actin, *HEX* hexachlorofluorescein, *RdRp* RNA dependent RNA polymerase.

### Real-time PCR

A real-time RT-PCR for BCoV detection was performed using previously described primers specific to the gene encoding the M protein^53^. Additionally, for internal control, a 200 µL mix of primers and probes specific to β-actin was prepared consisting of 5 µL of 100 µM ACT-1005-F and ACT-1135-R primers, 3.75 µL of 100 µM ACT-1081-HEX probe and 186.25 µL of water. The reaction was run in 20 µL of reaction mix that comprised 6.3 µL of water, 4 µL of 5 × QuantiTect Virus Master Mix (Qiagen, Hilden, Germany), 2 µL of both BCoV-F forward (10 µM) and BCoV-R reverse (10 µM) primers, 2 µL of BCoV-Pb probe (5 µM), 1.5 µL of a mixture of primers and probes specific to bACT, 0.2 µL of 100 × QuantiTect Virus RT Mix (Qiagen, Hilden, Germany) and 2 µL of RNA sample. After 30 min of reverse transcription at 42 °C and a 10 min incubation at 95 °C, 40 cycles of amplification were run each consisting of 15 s of denaturation at 95 °C and 45 s of annealing/elongation at 58 °C. As a positive control, for each PCR reaction, BCoV S379 Riems strain was used. All real-time PCR amplifications were performed using a LightCycler 96 Instrument (Roche, Mannheim, Germany). Sequences of primers used for real-time RT-PCR are presented in Table 2.

### Sanger sequencing

Samples positive in pancoronavirus RT-PCR were directly used for Sanger sequencing with Chu-RdRp-N2-F and Chu-RdRp-N2-R primers^52^. In the case of the samples positive in BCoV-specific real-time RT-PCR, they were amplified with a conventional RT-PCR using Sp1 and Sp2 primers specific to the conserved fragment of the S gene encoding the spike protein^53^ (Table 2). A Transcriptor One-Step RT-PCR kit (Roche, Mannheim, Germany) was used. The reaction was carried out in a total volume of 25 µL which included 1 µL of each primer (10 µM), 5 µL of 5 × reaction buffer, 0.5 µL of enzyme mix, 15.5 µL of PCR grade water and 2 µL of RNA sample. The amplification steps consisted of 30 min of reverse transcription at 50 °C followed by 2 min of incubation at 94 °C and 45 cycles consisting of 30 s of denaturation at 94 °C, 30 s of annealing at 55 °C and 30 s of elongation at 68 °C. The reaction was completed by a 10 min incubation at 68 °C. Specific 622-nucleotide-long products were visualized in 1.5% agarose gel. Positive samples were purified and used for Sanger sequencing with the Sp1 and Sp2 primers. Sanger sequencing was performed by Genomed SA (Warsaw, Poland). One partial sequence of the RdRp gene and one of the Spike protein gene were successfully sequenced and submitted to GenBank under accession numbers: PP263823 and PP263824 respectively. Both sequences originated from the nasal swab collected from the same free-ranging European bison from Borecka Forest in 2019. Phylogenetic trees were constructed by neighbour-joining using MEGA version 11^54,55^.

### Serology

Serum samples were tested for the presence of BCoV-specific antibodies using a semi-quantitative Monoscreen Ab Bovine coronavirus/Competition ELISA kit (Bio-X Diagnostics, Rochefort, Belgium). The assay was used in accordance with the instructions provided by the manufacturer.

### Statistical analysis

The chi-squared test was used to estimate associations between the proportion of positive samples and exposure variables such as age, sex, population type, sanitary status, and geographical origin. The confidence intervals were calculated using the exact binomial distribution.

### Ethics declaration

None of the European bison was samples for the purpose of this study. The samples were collected under the approval of the Minister of the Environment of 27 October 2014 (ZOP/06-061/51/2014); a decision of the general director for environmental protection (DZP—WG.6401.06.23.2014.km^2^) of 31 December 2014; and individual permits of the Director of the Białowieża National Park of 20 December 2013 (PN/061/22/2013) and 15 May 2017 (PN/061/14/2017). The monitoring was carried out in accordance with the authorizations of the Minister of Climate and Environment (DOP—1.61.21.2021.ZK) and the General Director of Environmental Protection (DZP—WG.6401.9.2021.EB).

## Data Availability

Sequence data that support the findings of this study have been deposited in the GenBank of National Center for Biotechnology Information (NCBI) under accession numbers PP263823 and PP263824. Additional data supporting the findings of this study are available from the corresponding author upon request.
